# Tissue micro array analysis of ganglioside *N*-glycolyl GM3 expression and signal transducer and activator of transcription (STAT)-3 activation in relation to dendritic cell infiltration and microvessel density in non-small cell lung cancer

**DOI:** 10.1186/1471-2407-9-180

**Published:** 2009-06-11

**Authors:** Hester van Cruijsen, Mariëlle Gallegos Ruiz, Paul van der Valk, Tanja D de Gruijl, Giuseppe Giaccone

**Affiliations:** 1 Department of Medical Oncology, VU University Medical Center, Boelelaan 1117, 1081 HV Amsterdam, The Netherlands; 2 Department of Pathology, VU University Medical Center, Boelelaan 1117, 1081 HV Amsterdam, The Netherlands; 3 Medical Oncology Branch, CCR, National Cancer Institute, NIH, Bethesda, MD 20892-1906, USA

## Abstract

**Background:**

Tumor immune escape and angiogenesis contribute to tumor progression, and gangliosides and activation of signal transducer and activator of transcription (STAT)-3 are implicated in these processes. As both are considered as novel therapeutic targets, we assessed the possible association of ganglioside GM3 expression and STAT3 activation with suppression of dendritic cell (DC) activation and angiogenesis in non-small cell lung cancer (NSCLC).

**Methods:**

Immunohistochemistry was performed on a tissue array to determine *N*-glycolyl GM3 (GM3) and phosphorylated STAT3 (pSTAT3) expression in 176 primary NSCLC resections. Median values of GM3 and pSTAT3 expression were used as cut off. Microvessel density (MVD) was determined by CD34 staining and morphology. CD1a and CD83 were used to determine infiltrating immature and mature dendritic cells, respectively.

**Results:**

94% and 71% of the NSCLC samples expressed GM3 and nuclear pSTAT3, respectively. Median overall survival was 40.0 months. Both low GM3 expression and high pSTAT3 expression were associated with a worse survival, which reached near significance for GM3 (*P *= 0.08). Microvessel density (MVD), determined by CD34 staining and morphology, was lower in NSCLC samples with high GM3 expression. CD1a^+ ^cells (immature DCs) were more frequent in NSCLC tissues as compared to peritumoral lung tissue, while CD83^+ ^cells (mature DCs) were more frequent in peritumoral lung tissue. CD83^+ ^DCs were less frequent in NSCLC tissues with high GM3 expression.

**Conclusion:**

GM3 and pSTAT3 are widely expressed in NSCLC. Based on CD83 expression, GM3, but not pSTAT3, appeared to be involved in tumor-induced DC suppression. pSTAT3 expression was not associated with MVD, while GM3 might play an anti-angiogenic role.

## Background

Lung cancer is the leading cause of cancer-related deaths worldwide. Non-small cell lung cancer (NSCLC), consisting mainly of adenocarcinoma, squamous cell and large-cell carcinoma, accounts for almost 80% of lung cancer cases. Five-year survival rate for NSCLC patients, irrespective of histological subtype and stage at diagnosis, approximates 15% [1]. Of the 25% who are candidates for curative surgery at diagnosis (stage I-IIIA), 65% will relapse within two years. Most patients present with advanced disease, and despite recent improvements in systemic combination regimens, advanced NSCLC patients still have a poor prognosis [2].

To design new and effective therapies in order to improve the outcome for NSCLC patients, understanding the tumor biology and the interplay between tumor cells and their micro-environment is of utmost importance. Some characteristics of tumor biology, like deregulated expression of gangliosides and constitutive activation of signal transducer and activator of transcription (STAT)-3, have been implicated in tumor-host interactions, i.e. tumor immune escape and angiogenesis. Tumor immune escape is established through a wide variety of active mechanisms employed by tumors to escape or to frustrate immune responses [3]. and angiogenesis is the formation of new blood vessels from the existing vasculature [4]. Both processes are employed by virtually all solid tumors to initiate and facilitate tumor progression.

Gangliosides are ubiquitous membrane-associated glycosphingolipids containing at least one sialic acid. Besides regulatory roles in normal physiological processes, gangliosides have been implicated in tumor development and progression [5]. The composition and production of gangliosides is altered in many tumour types. *N*-glycolyl GM3, a monosialic ganglioside, is not expressed in normal human tissues [6], but increased levels of *N*-glycolyl GM3 were detected in human breast tumors [7]. Gangliosides are also shed in the tumor microenvironment and eventually circulate in patients' blood [8,9]. These circulating gangliosides are thought to be involved in tumor-host interactions facilitating metastasis through tumor immune escape and tumor-associated angiogenesis. GM3 has been described to impair differentiation and function of both CD34^+ ^and CD14^+^-precursor derived dendritic cells (DC) [10,11]), which are the most potent antigen presenting cells essential for elicitation of an anti-tumor immune response, but are often hampered in their development and function by tumors [12]. In addition, maturation and function of Langerhans cells (i.e. DCs from the epidermis) were also shown to be inhibited by GM3 [13]. Not all studies, however, could establish such a DC immune suppressive role of GM3 [14]. Gangliosides can also modulate tumor-associated angiogenesis. Low GM3 levels relative to another ganglioside GD3 have been demonstrated to stimulate angiogenesis [15,16]. and GM3 was the only investigated ganglioside found not to increase endothelial cell responsiveness to the pro-angiogenic factor, vascular endothelial growth factor (VEGF) [17]. Although these observations suggest an anti-angiogenic role for GM3, the opposite has also been demonstrated: GM3 synergistically increased basic fibroblast growth factor (bFGF, another important pro-angiogenic factor)-induced proliferation of bovine aortic endothelial cells [18].

STAT-proteins are important in oncogenic signaling. This family comprises seven members: STAT1 to 4, STAT5a and STAT5b, and STAT6 [19]. Normally, STAT proteins transmit cytoplasmic signals from polypeptide cytokines or growth factors that have receptors with intrinsic or associated tyrosine-kinase activity, and consequently modulate the expression of target genes. Constitutive activation of STAT3 has been implicated in lung cancer development [20,21]. In addition, STAT3 activation through phosphorylation in tumor cells has been demonstrated to negatively regulate the adaptive immune responses both by reducing pro-inflammatory cytokine production, and by production of soluble factors inhibiting DC maturation [22]. Inhibiting STAT3 in tumor cells resulted in increased production of pro-inflammatory factors and reversed DC suppressive effects [22]. STAT3 activation has also been implicated in angiogenesis: STAT3 is a direct transcription activator of the *VEGF *gene and activation of STAT3 leads to tumor-associated angiogenesis *in vivo *[23].

More and more evidence is emerging for a relationship between angiogenesis and tumor-associated immune suppression. Many tumor-derived factors, such as interleukin-6, prostaglandins and VEGF, are implicated in both processes. In the present study we further set out to establish a possible role of GM3 expression and STAT3 activation in tumor immune escape and angiogenesis in NSCLC, since both are considered as novel therapeutic targets for this tumor type. To this end, we examined through immunohistochemistry the GM3 and phosphorylated STAT3 (pSTAT3) status of 176 primary NSCLC sections in a tissue micro array (TMA) and correlated this to DC infiltration, microvessel density and overall survival. We found that both GM3 and pSTAT3 are ubiquitously expressed in NSCLC. pSTAT3 expression could not be associated with either tumor immune escape or angiogenesis. In addition, we did not find evidence for a pro-angiogenic role of GM3, but we did observe an association of high GM3 expression levels with a decrease in the number of activated tumor-infiltrating DC in NSCLC patients.

## Methods

### Patients and NSCLC tissue micro array

**Figure 1:**
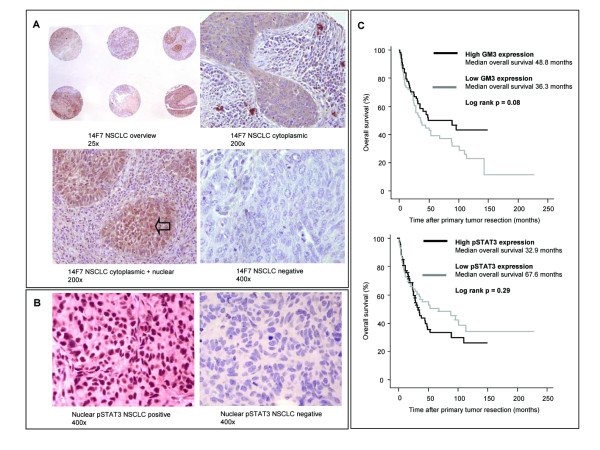
**GM3 and pSTAT3 expression.** (A) Overview of multiple NSCLC sections stained with 14F7 and examples of cytoplasmic, nuclear and negative staining of 14F7 in NSCLC. (B) Positive or negative nuclear pSTAT3 expression in NSCLC. Open arrows indicate example of nuclear staining; magnifications are indicated. (C) Kaplan-Meier survival analyses for overall survival based on GM3 expression or nuclear pSTAT3 expression in early-stage NSCLC patients. Median expression values were used to generate dichotomous variables. NSCLC, non-small cell lung cancer.

We studied 176 NSCLC patients, who underwent a primary tumor resection at the VU University Medical Center from 1988 until 2005. From the resected NSCLC material, tissue micro arrays (TMA) were created (Figure 1A) [24]. In short, paraffin-embedded tumor material was cut into 4 μm-thick sections and placed onto glass slides. Slides were stained with hematoxylin and eosin and an experienced pathologist verified the presence of tumor cells and marked the tumor area. 0.6 mm diameter biopsies were taken from the donor block, two from the tumor and one from the normal tissue area surrounding the tumor. Biopsies from the donor blocks were included in recipient tissue array blocks using a precision tissue array instrument (Beecher Instruments, Sun Prairie, WI, USA). From this tissue array block sections were made for immunohistochemistry.

Normal lung tissue, referred to as peritumoral lung tissue, was only available in 126 cases. As few biopsies mounted on the glass slide got lost during the procedure of creation and staining, in some analyses we did not obtain the maximum NSCLC samples of 176.

The study was approved by the Medical Ethical Committee and carried out in accordance with the ethical guidelines of our institution concerning informed consent about the use of patient's material after surgical procedures.

### Immunohistochemistry

TMA sections were deparaffinized and endogenous peroxidase activity was blocked with 0.3% hydrogen peroxide/methanol for 30 minutes. TMAs were subjected to none, 1 mM Tris/EDTA- or 10 mM citrate-based antigen-retrieval, depending on which primary antibody was used. Subsequently, slides were incubated with the appropriately diluted primary antibody at 4°C in a moist chamber. Bound primary antibodies were visualized using EnVision-reagents and diaminobenzidine (DAB+) chromogen (DakoCytomation, Glostrup, Denmark). Slides were counterstained with haematoxylin. Scoring and quantitation for all stainings were based on the whole biopsy surface (0.28 mm^2^) and on consensus between two independent observers.

To determine *N*-glycolyl GM3 expression in NSCLC tissue, the murine IgG1 anti-GM3 (*N*-glycolyl) monoclonal antibody 14F7 was used (kindly provided by Daniel Alonso, Buenos Aires, Argentina) [7]. Immunostaining with 14F7 did not require antigen retrieval, and 14F7 (1:1000) was incubated for 18 hours at 4°C. Mean percentage 14F7 positive cells of total number of tumor cells were determined per NSCLC case. In most statistical analyses, median value was used to create dichotomous variables.

STAT3 activation was determined by staining with a monoclonal antibody against STAT3 phosphorylated at tyrosine residue 705 (Clone D3A7; Cell Signaling, Boston, MA). Pre-treatment involved heating the slides in Tris/EDTA for 30 minutes. The primary antibody (1:50) was then incubated overnight at 4°C. NSCLC samples with nuclear expression of pSTAT3 were designated positive. Number of positive tumor cells was multiplied by intensity of staining (0, 1, 2, or 3). Dichotomous variables were generated using median values as cut off.

Immature and mature DC infiltration was determined by monoclonal antibodies against CD1a (Clone MTB1; Monosan, Uden, The Netherlands) and CD83 (Clone 1H4b; Monosan, Uden, The Netherlands), respectively [25]. Both CD1a and CD83 immunostaining required citrate-based antigen retrieval. Both CD1a (1:5) and CD83 antibodies (1:25) were incubated for 1 hour at 4°C. Mean number of CD1a or CD83 positive cells per NSCLC case or per corresponding peritumoral lung tissue was calculated. If any infiltrating immature or mature DC were present, NSCLC cases were designated positive for immature or mature DC infiltration.

Microvessel density (MVD) per section was measured using immunostaining with a CD34-monoclonal antibody (Clone QBEnd10; DakoCytomation, Glostrup, Denmark), which required citrate-based antigen retrieval and an one-hour incubation of the primary antibody (1:50) at 4°C. MVD of each NSCLC case was defined as the mean number of CD34^+ ^vessels per section (0.28 mm^2^). Since the sections were small, we did not identify a hot spot to count the CD34^+ ^vessels, but counted vessels in the entire section.

### Statistics

The statistical analysis between categorical data was done using the Pearson's Chi-Square test. Student's t-tests were performed to compare categorical data with continuous data. Kaplan-Meier plots and log rank analysis were applied to determine the significance of differences in overall survival. Values of *P *≤ 0.05 (two-tailed) were considered statistically significant.

Overall survival was the time between diagnosis and the date of death or the date at which patients were last known to be alive. For survival analysis, data collection was locked on 21^st ^of February, 2007.

## Results

### Patient characteristics

NSCLC samples of 176 patients, who underwent resection of their primary tumor, were included on the TMAs. A summary of patient characteristics is listed in Table 1. Median overall survival of the population was 40.0 months. Low tumor stage and complete resection were predictive of favourable prognosis (log rank, *P *= 0.02 and *P *= 0.0009, respectively). Age, gender and histological subtype were not correlated with overall survival.

**Table 1:** Patient characteristics

**Mean age (years)**	64.5
**Gender (%)**	
Male	72
Female	28
**Histology (%)**	
Adenocarcinoma	36
Squamous cell carcinoma	43
Large cell carcinoma	15
Adenosquamous cell carcinoma	3
Broncho-alveolar carcinoma	4
Stage (%)*	
I	51
II	29
III	20
**Smoking history (%)**	
Current	34
Former	39
Non-smoking	2
Unknown	25

* According to American Joint Committee on Cancer

### GM3 and pSTAT3 expression in NSCLC

GM3 was expressed in almost all NSCLC samples: 94% of the 165 evaluable NSCLC samples expressed GM3, i.e. more than 5% of the tumor cells were positive. We defined positive cells as having cytoplasmic staining. Furthermore, GM3 expression was high: in 52% of all NSCLC samples, over 90% of the tumor cells were positive for 14F7. In 15% of the cases additional nuclear staining of 14F7 could be detected (Figure 1A). No association could be established between the different histological subtypes of NSCLC and GM3 expression, nor between tumor stage and GM3 expression. Of note, the GM3 expression rate in peritumoral lung tissue samples was also high, i.e. of 97% of the peritumoral samples, more than 5% of the lung epithelial cells were positive. However, expression levels were significantly lower than in NSCLC samples: in only 19% of all peritumoral lung samples, over 90% of the tumor cells were positive for 14F7 (as compared to 52% in NSCLC, peritumoral vs NSCLC, *P *< 0.001).

pSTAT3 expression in the nucleus was present in 71% of the 164 evaluable NSCLC samples. Typical nuclear pSTAT3 immunostaining in NSCLC is shown in Figure 1B. Although previously reported to be associated with smaller tumors, limited smoking history and adenocarcinoma [20], we could detect no such associations between nuclear pSTAT3 expression and the clinical parameters tumor stage, histology, and smoking history. Peritumoral pSTAT3 expression did not differ significantly from pSTAT3 expression in NSCLC.

Using the median value as cut off, NSCLC patients with low GM3 expression (median percentage of GM3-positive cells, 91%) or high pSTAT3 expression (median number of pSTAT3 positive cells multiplied by intensity of staining [see Materials and Methods], 110) tended to have a worse overall survival, although this did not reach statistical significance (Figure 1C).

### DC infiltration and GM3 or pSTAT3 expression in NSCLC tissue

**Figure 2:**
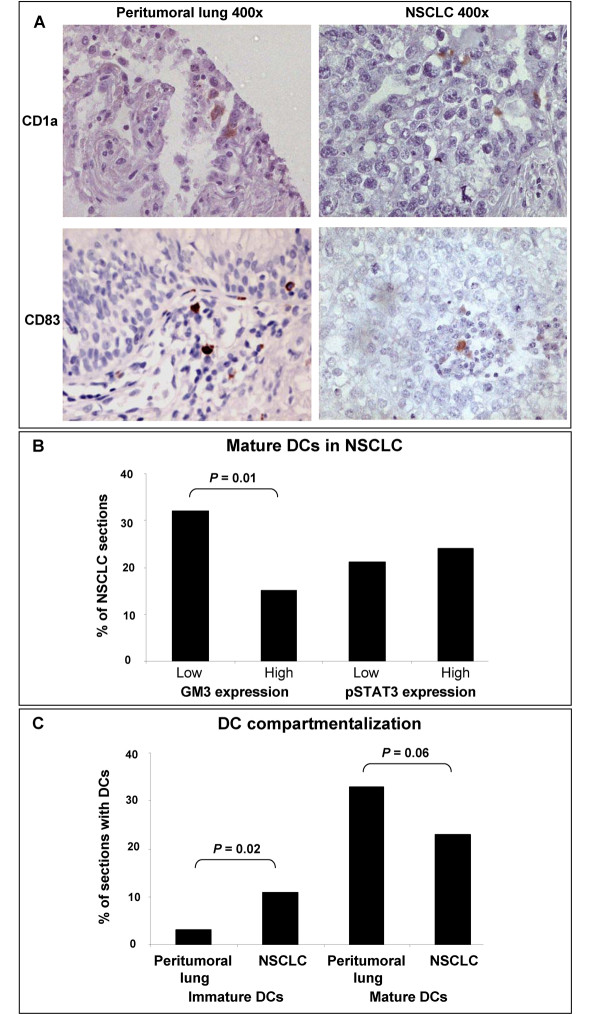
**CD1a and CD83 expression.** (A) CD1a and CD83 immunostaining in both NSCLC tissues and peritumoral lung tissues. Magnifications are indicated. (B) Mature (i.e. CD83^+^) DC infiltration in NSCLC with low (below median) or high (above median) GM3 or pSTAT3 expression. (C) Compartmentalization of immature (CD1a^+^) and mature (CD83^+^) DCs as determined by their infiltration in peritumoral lung tissue or NSCLC tissue. NSCLC, non-small cell lung cancer.

Typical immunostainings of CD1a and CD83 in NSCLC and peritumoral lung tissue are shown in Figure 2A CD1a^+ ^DC infiltration and GM3 expression status in NSCLC were not found to be associated (data not shown). Mature CD83^+ ^DCs, however, were significantly more often present in NSCLC samples with low GM3 expression as compared to NSCLC samples with high GM3 expression (Figure 2B). Presence of either immature or mature DCs was not associated with nuclear pSTAT3 expression in NSCLC. In addition, DC numbers were not associated with tumor stage, histology, or smoking history and we could not establish any association between the number of infiltrating CD1a^+ ^or CD83^+ ^DCs and overall survival of NSCLC patients.

The compartmentalization of DCs in tumoral versus peritumoral tissues was previously associated with differential DC maturation status in breast and colorectal carcinomas [25-27]. Since TMAs contain small sections (0.28 mm^2^), exact architecture of the NSCLC tissue and the tumor-associated stroma is often lost. To investigate whether the (peri-)tumoral localization of DC is correlated with its maturation status, we therefore compared the presence of immature (CD1a^+^) and mature (CD83^+^) DC in the NSCLC samples with their incidence in peritumoral lung sections. Although overall frequencies of both immature and mature DCs were low, we found an increased number of immature CD1a^+ ^DCs in NSCLC tissue as compared to peritumoral lung tissue (Figure 2C). Consistently, we observed less mature CD83^+ ^DCs in NSCLC tissue as compared to peritumoral lung tissue (Figure 2C), which was in keeping with a previously observed compartmentalization according to DC maturation status [25-27].

### Microvessel density and GM3 or pSTAT3 expression in NSCLC tissue

**Figure 3:**
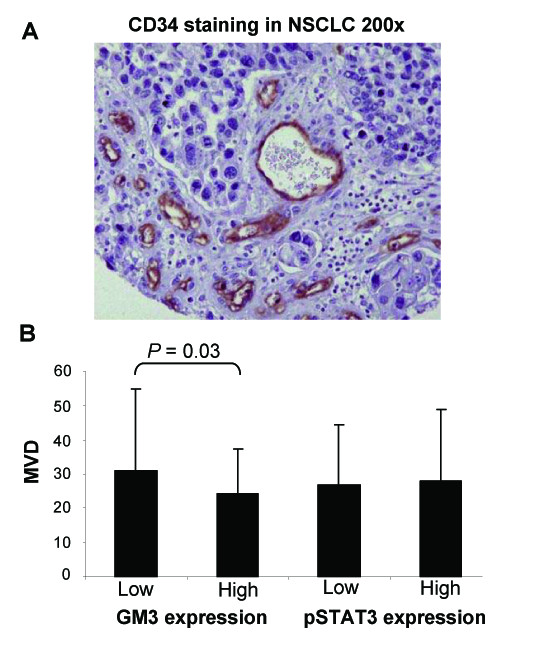
**Microvessel density.** (A) CD34 immunostaining of vasculature in NSCLC to determine microvessel density. Magnification is indicated. (B) Microvessel density (MVD) according to low (i.e. below median) or high (i.e. above median) GM3 expression or pSTAT3 expression in NSCLC samples. Mean MVD values and SD are shown. NSCLC, non-small cell lung cancer.

MVD was defined as the mean number of CD34^+ ^vessels per section of one NSCLC sample (Figure 3A). MVD in NSCLC was not associated with tumor stage and smoking history. Conflicting reports have been made about the association between MVD and histology [28-30]. We found a significantly higher MVD in adenocarcinoma as compared to squamous cell carcinoma (mean values per NSCLC case [SD]: adenocarcinoma 32.2 [26.2]; squamous cell carcinoma 22.6 [11.4], *P *= 0.02). MVD was not found to be associated with overall survival of NSCLC patients.

NSCLC samples with low GM3 expression had a slightly, but significantly, higher MVD as compared to high GM3-expressing NSCLC samples (Figure 3B), while MVD was not significantly different between NSCLC with low or high nuclear pSTAT3 expression (Figure 3B).

## Discussion

Our studies show that both GM3 and pSTAT3 are widely expressed in NSCLC and that GM3 expression is associated with a favourable patient survival (*P *= 0.08). To date, no reports have been made about the prognostic value of GM3 expression, while pSTAT3 has previously been demonstrated not to be associated with survival in NSCLC patients [20,31]. In our patient population, we found a trend for an association between high pSTAT3 expression in NSCLC and worse prognosis, although this did not reach significance (*P *= 0.29).

In literature contradictory reports have been made about the anti-angiogenic or pro-angiogenic potential of GM3 [15-17]. We found no evidence for a pro-angiogenic role, but rather the opposite, since GM3 expressing tumors were observed to have a slightly lower MVD. Gangliosides are biosynthesized by sequential enzymatic modifications by sialyltransferases; GM3 can be converted into GD3 by alpha2,8-sialyltransferase [5]. As low GM3 levels relative to GD3 has been implicated in angiogenesis [15], GD3 rather than GM3 may be the active pro-angiogenic factor. The next issue to be addressed should therefore be whether alpha2,8-sialyltransferase is expressed in the NSCLC cases with low GM3 expression and whether this coincides with high GD3 expression. GD3 in turn may then be correlated to high MVD in contrast to GM3. Further functional studies are clearly needed to confirm the distinct roles of GM3 and GD3 in angiogenesis.

pSTAT3 expression has been implicated in tumoral VEGF production and angiogenesis [23]. We could not establish a correlation between MVD and nuclear pSTAT3 expression in NSCLC. However, since the correlation between VEGF and MVD in NSCLC remains controversial [30], the lack of the correlation between pSTAT3 expression and MVD may not be unexpected. We did not observe a difference in nuclear pSTAT3 expression between peritumoral and NSCLC tissue. The lack of this difference might result from the production of cytokines and growth factors (like interleukin-6 and VEGF) by tumor cells. These factors might condition the surrounding cells, resulting in translocation of activated pSTAT3 to the nucleus in peritumoral non-malignant tissues.

Although the frequencies of both CD1a- and CD83-positive cells were low, we observed an inverse correlation between GM3 expression and infiltrating mature DCs, in keeping with previous reports, which showed that GM3 inhibited DC differentiation, maturation and migration [10,11,13]. GM3-induced inhibition of DC maturation and migration may have led to less mature DCs infiltrating the tumoral tissue, and consequently, may have contributed to the observed compartmentalization of DCs according to their maturation status: consistently, we observed cells expressing CD1a, which is generally believed to be a marker of immature DCs and to be down-regulated upon maturation, more frequently in NSCLC tissue as compared to the corresponding peritumoral lung tissue. In addition, we observed cells positive for CD83, a DC maturation marker, more frequently in the peritumoral lung tissue as compared to the NSCLC sections.

Although pSTAT3 expression in tumor cells was reported to be associated with production of DC suppressive cytokines [22], DCs of any maturation status were not differentially infiltrated in NSCLC samples with high pSTAT3 expression as compared to NSCLC samples with low pSTAT3 expression.

Although some reports have suggested that infiltrating mature DCs predict a favourable prognosis in NSCLC [32,33], we could not establish a correlation between high numbers of mature tumor-infiltrating DCs and patient survival. The general paucity of infiltrating DCs in the relatively small NSCLC sections examined on the TMAs might explain the observed lack of correlation between DC infiltration and patient survival. More data have been published about the prognostic significance of MVD in NSCLC, and although many reports suggest that higher MVD predicts an adverse prognosis, the exact prognostic value of MVD remains unclear [30,32]. We could not establish any correlation between MVD in NSCLC tissue and patient survival. Differences in technical procedures might make our results on MVD hard to compare with published data. Since only small NSCLC sections (0.28 mm^2^) were mounted on the TMA glass slides, structure of the tumor-microenvironment might have been lost. Scoring microvessels might be underestimated in these small sections, especially when considered that MVD is normally evaluated in the most intense vascularization areas of the tumor stroma [32,34]. Although TMAs are suitable for studying tumor cell characteristics, they may not be applicable when studying tumor-associated angiogenesis. On the other hand, one could argue that TMAs with a fixed size might be more appropriate to score MVD, since scoring MVD in the most intense vascularization area of the tumor stroma is susceptible to investigator's bias.

Our observations of comparable levels of pSTAT3 between healthy and tumor tissues and a lack of correlation with MVD or mature DC infiltration do not support the implementation of STAT3 inhibitors as anti-angiogenic or immunostimulatory therapeutics in NSCLC. In contrast, GM3 was expressed at significantly higher levels in NSCLC than peritumoral lung tissues and was associated with tumor-related DC suppression, making this an attractive target for anti-cancer therapies. Approaches to target gangliosides are currently in early clinical development. Many studies focus on monoclonal antibodies including antibodies against *N*-glycolyl-containing gangliosides (i.e., 1E10) and on vaccine-based strategies [35-38]. Although induced immune responses have been reported, data on clinical efficacy are still lacking. The more specific murine monoclonal antibody 14F7, which we used in this study and recognizes *N*-glycolyl GM3, has entered clinical development [39]. Further studies are awaited to establish the clinical efficacy of humanized 14F7. Our present data suggest that side studies monitoring the immune status could be of additional value to define the role of GM3 in tumor immune escape, e.g. through DC suppression.

## Conclusion

Our immunohistochemical TMA studies show that GM3 and pSTAT3 are widely expressed in NSCLC. pSTAT3 expression could not be associated with either DC-based tumor immune escape or angiogenesis, negating its suggested role as a 'master-switch' in these tumorigenic processes, at least for stage I-III NSCLC. In addition, we did not find evidence for a pro-angiogenic role of GM3. We could, however, confirm a possible involvement of GM3 in tumor-induced DC suppression in NSCLC patients. Its high tumor-specific expression makes GM3 a possible candidate for tumor targeting. Our findings suggest that it might be worthwhile to also monitor any angiogenic and immune effects of these therapeutic strategies that are currently under clinical development.

## Competing interests

The authors declare that they have no competing interests.

## Authors' contributions

HvC participated in the design of the study, carried out the immunohistochemistry studies, collected, analyzed the data, and drafted the manuscript. MGR participated in collecting the data. PvdV helped in collecting and analyzing the data. TdG helped interpreting the data and has been involved in the design of the study and drafting the manuscript. GG initiated the study and participated in its design and coordination. All authors read and approved the final manuscript.

## Pre-publication history

The pre-publication history for this paper can be accessed here:

http://www.biomedcentral.com/1471-2407/9/180/prepub
